# Training the Sight

**Published:** 1898-03-05

**Authors:** 


### Training the Sight.

At the Society of Arts, on the 23rd inst., Mr. Brudenell Carter read a paper on the sight of children in schools, in which he took occasion to repeat advice which, if we remember rightly, he has given more than once before, namely, that sight should not be regarded as a power or faculty which may be trusted to take care of itself, but that it should be systematically trained by the aid of appropriate objects, and that its quality should be tested from time to time. It has long been found necessary, in the public services and in some railways, to adopt a standard of vision which must be possessed by candidates for employment; and this standard rests upon the power to decipher capital letters, of such a size and placed at such a distance, that they subtend a visual angle of five minutes at the eye of the observer. All our conceptions of the size of an object are founded partly upon the size of its retinal image, and partly on what we conceive to be the distance of the object itself. The power to see the shape and outline of an object depends upon the image being of such a size as to leave unaffected nerve fibres in areas over which the outline of the object itself does not extend; in such a way, for example, that the image upon the retina formed by a capital E must be large enough for the two spaces between its three horizontal limbs to be each actually wider than the diameter of a nerve fibre, and thus to be occupied by fibres upon which no part of the image falls. As soon as the image becomes so small that the spaces referred to are smaller in diameter than single nerve fibres, none of the latter will be left unaffected, and the spaces themselves will cease to be recognisable by vision. The power to distinguish letters under a visual angle of five minutes, although commonly accepted as affording the standard of normal vision, is constantly surpassed by individuals, and even by races of mankind. Travellers have told marvellous stories about the sight of many savage tribes, and, although some of these stories may be received with incredulity, while others are explicable by the greater clearness of the atmosphere in the countries where they occurred; yet there seems no sufficient ground for doubting that the visual function, like every other, is capable of being improved by judicious exercise, and especially by such exercise as is afforded by the necessity of reliance upon its faintest indications. There is no reasonable doubt that the Siberian Tartar, who, when looking at Jupiter, told Arago that he had seen the big star swallow a little one and spit it out again, had really seen, with his unaided eyes, an occultation of the third satellite. Sir H. Truman Wood, in the discussion at the Society of Arts, spoke of an Englishman who could see some of the double stars; but it must be remembered that the Englishman could easily know what stars were double, and could, perhaps, fancy that he saw their peculiarity; while the Tartar could have had no knowledge of the very existence of the satellite if he had not seen it. Mr. Carter maintained that the habit of seeing as much as possible, of earnest visual attention to the details of the environment, would certainly have the effect of increasing the activity of the visual function, and also, in all probability, of promoting the growth of finer fibres in the retina, by the aid of which smaller images could be appreciated. He gave reasons for believing that the vision of town-bred children is less acute than that of the country-bred; the former seeing chiefly large objects, such as houses and omnibuses, under large visual angles; the latter habitually attending to smaller or more distant objects, and using the eyes under smaller visual angles. The general moral of the discourse was that all school teachers should be instructed to test the vision of new pupils, and to record the facts in a register, at the same time calling the attention of parents to cases of manifest defect, and thus enabling them to obtain timely advice, or to regulate the course of education with reference to the special requirements of each child. It was further suggested that vision should be trained in schools by the use of difficult test objects set at proper distances; it being only work upon near objects that is ever injurious to the eyes, while work upon distant objects must always be of the kind by which the faculty exercised is likely also to be improved. It was maintained that sight might even be permitted to take its place among the physical qualities that are made the bases of competitions, and that prizes might be awarded for excellence. It seems certain that there are positions in life in which the power to see acutely might be quite as valuable as, or even more valuable than, the power to run swiftly; and there seems no valid reason why the recognition which is daily given to the latter should not be extended also to the former.

